# Walnut By-Products and Elderberry Extracts—Sustainable Alternatives for Human and Plant Health

**DOI:** 10.3390/molecules29020498

**Published:** 2024-01-19

**Authors:** Anca Sandu-Bălan (Tăbăcariu), Irina-Loredana Ifrim, Oana-Irina Patriciu, Ioana-Adriana Ștefănescu, Adriana-Luminița Fînaru

**Affiliations:** 1 Doctoral School in Environmental Engineering, “Vasile Alecsandri” University of Bacau, 157 Marasesti Str., 600115 Bacau, Romania; anca_tabacariu@yahoo.com; 2 Department of Chemical and Food Engineering, “Vasile Alecsandri” University of Bacau, 157 Marasesti Str., 600115 Bacau, Romaniaadrianaf@ub.ro (A.-L.F.)

**Keywords:** walnut, elderberry, extracts, green chemistry, biological activities, phytosanitary properties

## Abstract

A current alternative for sustainable development through green chemistry is the replacement of synthetic compounds with natural ones through the superior capitalization of natural resources, with numerous applications in different fields. The benefits of walnuts (*Juglans regia* L.) and elderberries (*Sambucus nigra* L.) have been known since ancient times, due to the presence of phytochemicals such as flavonoids, polyphenols, carotenoids, alkaloids, nitrogen-containing compounds, tannins, steroids, anthocyanins, etc. These active compounds have multiple biological activities for human health, including benefits that are antibacterial, antioxidant, anti-inflammatory, antidiabetic, hepatoprotective, antihypertensive, neuroprotective, etc. Like other medicinal plants, the walnut and the elderberry possess important phytosanitary properties (antibacterial, antifungal, and insecticidal) and their extracts can also be used as environmentally safe biopesticides, with the result that they constitute a viable and cheap alternative to environmentally harmful synthetic products. During recent years, walnut by-products and elderberries have attracted the attention of researchers, and investigations have focused on the species’ valuable constituents and active properties. Comparing the information from the literature regarding the phytochemical profile and biological activities, it is highlighted that, apart from the predominant specific compounds, the walnut and the elderberry have common bioactive compounds, which come from six classes (phenols and derivatives, flavonoids, hydroxycinnamic acids, tannins, triterpenoids, and phytosteroids), and act on the same microorganisms. From this perspective, the aim of this review is to provide an overview of the bioactive compounds present in the different constitutive parts of walnut by-products and elderberries, which present a specific or common activity related to human health and the protection of agricultural crops in the context of sustainable development.

## 1. Introduction

Maintaining the health of the population, as well as the quality of the environment in which we live, requires the correct management of economic relations, social resources, and, even more importantly, everything that can be considered as natural resources.

In this regard, special interest is given to plants that have been and are used in traditional medicine due to the rich content of their different compounds with bioactive properties. In significant proportions, plants are used in the pharmaceutical and nutritional supplement industry as primary sources for the extraction of natural compounds with beneficial effects, especially for preventive but also therapeutic purposes [1,2,3,4].

Among the fundamental needs of modern industry, because of overexploitation and globalization, are sustainability and the so-called “green chemistry”, which involves replacing synthetic compounds with natural ones from plants, in order to capitalize and develop natural extracts, with numerous applications in different fields, such as plant culture, the pharmaceutical industry, food, textiles, the cosmetic industry, etc. [5].

The walnut (*Juglans regia* L.) has been cultivated in Romania since ancient times, and it is known that the Geto-Dacians used walnut oil in their diet. Developed over the centuries, this tradition has propelled Romania to being among the largest walnut-producing countries in the world. Until 1900, the walnut occupied large areas of forests and plantations on the forest floor [6]. Romania remains one of the largest producers of walnuts in the European Union, according to data published by the European Commission. For 2021, Eurostat data indicate a walnut production in Romania of 60,820 tons [7]. In 2022, approximately 2200 ha of walnuts were harvested in Romania [8]. According to the National Institute of Statistics’ 2022 report, it is noteworthy that, regarding the regional distribution, in the northeast region of the country, the areas of Bacău, Iași, and Suceava produce the largest quantities (1703–1873 tons/year). Significant productions are also found in the areas of Buzău and Vrancea (in the southeast region) and Bihor and Maramureș (in the northwest region), according to the same report [9]. There is also a major interest in the development of sustainable organic walnut production throughout Romania [10,11]. 

At the same time, the elderberry has an abundant presence in the spontaneous flora of Romania, especially in the northeast region of the country, and can represent a valuable and cheap resource for bioactive principles. The most widespread species here (there are around 20 in total) is the black elder (*Sambucus nigra* L.). The specialized literature also talks about three autochthonous varieties: Ina, Nora, and Brădet. Being a humidity-loving species, in the spontaneous flora, the elderberry grows on land with groundwater on the surface (on terraces of flowing waters) or on the edge of forests, generally in more shaded areas [12,13,14]. 

Various studies have shown that walnuts and elderberries, which are popular all over the world, are two medicinal plants that, thanks to their phytochemical compounds (proteins, flavonoids, phenolic acids, polyphenols, vitamins, and minerals), have beneficial properties for the human body (antibacterial, antioxidant, antiviral, analgesic, anti-inflammatory, etc.) and also possess phytosanitary properties (antibacterial, antifungal, and insecticidal) with benefits on other plants [12,13,14,15,16,17,18].

The purpose of this review is to highlight the importance of natural compounds from walnut by-products and elderberry: their biological activities in terms of human health and their phytosanitary properties that could also include the potential for the protection of agricultural crops, respectively. In this context, the available data regarding the predominant bioactive compounds specific to each species and, on the other hand, the common phytochemicals that act on the same microorganisms were critically examined and synthesized, suggesting, in the context of sustainable development, the possibility of exploiting them in a mixture of walnut by-products and elderberry extracts.

## 2. Walnut (*Juglans regia* L.) and Elderberry (*Sambucus nigra* L.) Characterization

Due to the great diversity of extraction and analysis methods, there are numerous studies in the scientific literature that indicate both the chemical composition of walnuts and elderberries and their biopharmaceutical properties and applications in various fields.

### 2.1. General Description

**Figure 1:**
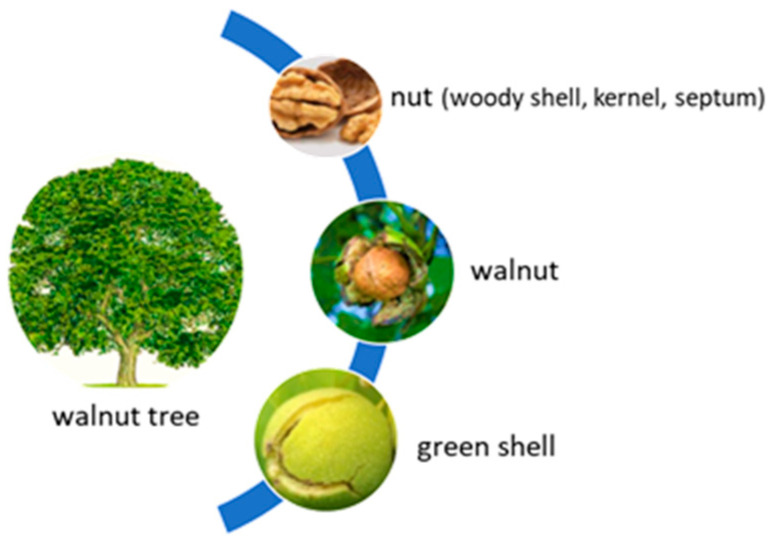
The main components of the walnut.

*Juglans regia*, a tree belonging to the Juglandaceae family, is also known as the common walnut [19]. The walnut tree is a vigorous tree that can reach 30 m in height, has strong branches, and has a very wide and rich crown. The leaves are large, composed of 5–9 elliptical leaflets, with whole margins. The fruit includes four main parts: the kernel, the septum, the woody shell, and the green shell (Figure 1). The kernel is the edible part of the fruit, being widely consumed by humans. The other parts of the walnut (the green shell, the dry woody shell, the septum, the bark, the branches, and the leaves) are used for various purposes [16,20].

The green shell is used in traditional Chinese medicine to fight cancer due to its antioxidant properties, as well as for the treatment of pain and for inflammation [21]. The leaves are used to relieve minor inflammatory skin disorders [22]. The walnut shell that results from the processing of walnuts through a successful process of valorization can be another source of active compounds [23,24]. The ripe nut fruit is used in confectionery, while the young form of the fruit is mainly used in the production of liquor [25]. 

**Figure 2:**
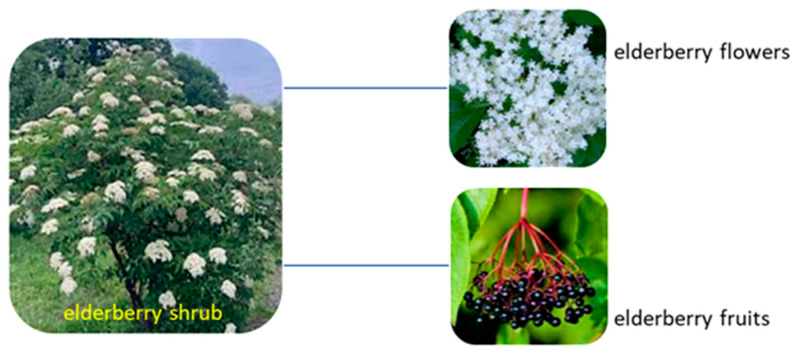
The component parts of elderberry.

The elder, black elder, or elderberry (*Sambucus nigra* L.) is used as a medicinal and ornamental plant. In the Middle Ages, among the ancient Germans, the elderberry was considered a holy plant, also being used in traditional medicine [12]. In popular tradition, the elderberry, a species of plants in the group of shrubs in the Adoxaceae family (Figure 2), is considered the number one antiviral medicine [13,14]. The hermaphrodite flowers are fragrant, while the leaves have an unpleasant odor when rubbed in the hands. These flowers are followed by clusters of small black fruits. Elderberries are a powerful source of essential vitamins and minerals [13]. 

### 2.2. Chemical Composition

The chemical composition varies within very wide limits due to factors related to the species, the variety, pedoclimatic conditions, the harvest period, the degree of ripening, and extraction conditions, as well as the analysis and identification methods. 

#### 2.2.1. Chemical Composition of Walnut 

The benefits of the walnut, which have been known since ancient times, are due to the presence of some bioactive compounds such as flavonoids, polyphenols, juglones, tannins, steroids, etc. [15,16,17,26].

- Chemical composition of the walnut kernel

The walnut kernel, which mainly has economic importance, contains 4% water, 65% fats, 15% proteins, and 14% carbohydrates, vitamins, and minerals [16].

As described by Slatner et al. [27], who in his work used 2.5 g of ground walnut kernel mixed with 22.5 mL of CH_3_OH/H_2_O (*v*/*v*, 60/40) in a chilled water bath (0 °C) with sonication for 60 min and analyzed extracts via HPLC, and also in other studies [28,29,30,31,32,33,34], the main bioactive compounds present in walnut kernel are tannins (glansreginin A and ellagic acid derivatives), flavonoids (procyanidin trimer), γ-tocopherol (vitamin E), steroids (β-sitosterol), triterpenes, and fatty acids (Table 1).

- Chemical composition of walnut by-products

The extracts from walnut by-products contain a variety of useful compounds compared to the extracts from the walnut kernel. 

##### Chemical Composition of Walnut Leaves

According to Medic et al. [35], who performed the extraction of 0.25 g of leaf powder with methanol in an extract ratio of 1:30 (*w*/*v*) leaves: methanol using ultrasonication for 60 min and analyzed the extracts via HPLC–MS, and other research [36,37,38,39,40,41,42], walnut leaves contain a large amount of compounds from the class of hydroxycinnamic acids (neochlorogenic acid and 3-p-coumaroylquinic acid), flavonoids ((+) catechin, procyanidin dimers, and quercetin derivatives), and naphthoquinones (hydrojuglone derivatives, juglone, and sdihydroxytetralone hexoside) (Table 2).

##### Chemical Composition of Walnut Shell

Green walnut shell extracts can be obtained using several extraction methods. For example, the green walnut shell extract can be prepared through maceration by stirring the raw material (1 g) in an aqueous ethanol solution (80% ethanol, *v*/*v*; 30 mL) at 25 °C for 60 min [43]. Ultrasonic assisted extraction (UAE) and supercritical CO_2_ extraction were used to increase the extraction yield. For the UAE, the optimal conditions were a temperature of 60 °C, an extraction time of 30 min, and a mixture of 60% ethanol–water [20]. For the supercritical CO_2_ extraction, the optimal conditions were established as 68 °C and 20% ethanol [44]. The total phenol content was determined with the Folin–Ciocâlteu method, using phenolic acids and juglone with ultra-high performance liquid chromatography (UHPLC) [45]. Jahanban-Esfahlan et al. [20] presented a summary of the compounds present in the green walnut shell extract.

Furthermore, the dry walnut shell contains compounds from the class of hydroxybenzoic acid derivatives, sterols, mono- and triglicerides, minerals and (calcium, potassium, and iron). For example, Queirós et al. [46] used 2 g of dry crushed walnut shell for Soxhlet extraction with different solvents (dichloromethane, ethanol, and water) and performed the extract analysis via a UV-coupled high-pressure ion exclusion chromatography (HIPCE-UV). 

In agreement with this study and others [43,47,48,49], the walnut shell contains the following bioactive compounds from the classes of naphthoquinones (juglone and 1,4-naphthoquinone), tannins (tannic acid), hydroxybenzoic acids, hydroxycinnamic acids (sinapic acid and rosmarinic acid), and flavonoids ((−) epicatechin, myricetin, and apigenin) in larger amounts (Table 3). It may also be noted that the walnut shell contains six times more juglone than walnut leaves.

##### Chemical Composition of the Walnut Septum

As reported by Genovese et al. [50], after the extraction of the walnut septum through ethanolic maceration (10 g of powdered walnut septum in 50 mL of 96% ethanol for 48 h at room temperature) and analysis via HPLC with an ionization mass spectrometry (HPLC/ESI-MS), and several other studies [51,52,53,54], the walnut septum contains an important amount of compounds from the classes of tannins (ellagic acid) and flavonoids (quercitrin) (Table 4).

#### 2.2.2. Chemical Composition of Elderberry

The chemical composition of the elderberry indicates the presence of many bioactive substances [12,13,14]. Raw elderberries contain, per 100 g, 18.4 g of carbohydrates, 7 g of fiber, 0.5 g of fat, 0.66 g of protein, 30 μg of vitamin A, 0.07 mg of thiamin (B1), 0.06 mg of riboflavin (B2), 0.5 mg of niacin (B3), 0.14 mg of pantothenic acid (B5), 0.23 mg of vitamin B6, 36 mg of vitamin C, and minerals (calcium—38 mg; iron—1.6 mg; magnesium—5 mg; phosphorus—39 mg; potassium—280 mg; and zinc—0.11 mg) [55,56,57]. The fruits of the Sambucus tree also contain rutin and isoquercetin; anthocyanins; acids (citric, quinic, and malic); tannins; volatile oils; and mucilage. Elderberries that are fully ripe, which are very attractive from a nutritional point of view, can be consumed as such or in juice form. The elderflowers contain rutoside (rutin) (3%), small amounts of volatile oil (0.03%), sugars, vitamin C, etc. In the elder bark, the presence of choline, sugars, and tannins were observed. The leaves of elder contain glycolic aldehyde, oxalates, appreciable amounts of vitamin C, etc. Sambunigrin, a cyanogenic glycoside, which is missing in the other species of the genus, is present in the leaves, bark, seeds, flowers, and unripe fruits [58,59].

- Chemical composition of elderberry leaves

According to several studies [12,13,14,18,58,59,60,61,62,63], elderberry leaves contain a large amount of phenolic compounds (caffeic acid and chlorogenic acid), flavonoids (quercetin-rutinoside), and anthocyanins (cyanidin-glucoside) (Table 5). For example, Kiprovski et al. [62] studied the changes that occurred during ripening in the leaves and fruits of wild and cultivated *Sambucus nigra* through sonication and analyzed the extracts using HPLC-DAD-ESI-MS.

- Chemical composition of elderberry flowers

Vujanović et al. [64], after performing the extraction through different methods (maceration, sonication, and microwave-assisted extraction) using different solvents and analyzing the extracts using the LC–MS/MS technique, found that microwave-assisted extraction provides the highest number of bioactive compounds. According to this study and others [18,58,60,64,65], the elderberry flowers contain a large amount of compounds from the classes of phenols (quinic acid), flavonoids (naringenin, 5-*O*-caffeoylquinic acid, and rutin), and terpenes (ursolic acid) (Table 6).

- Chemical composition of elderberries

Avula et al. [66], Kiprovski et al. [62], Salvador et al. [67], Pinho et al. [60], Sidor and Gramza-Michałowska [18], Mocanu and Amariei [12], Pascariu and Israel-Roming [13], and Haș et al. [14] showed that Sambucus fruits contain a number of phytochemical compounds, such as phenolic acid (chlorogenic acid), flavonoids (quercetin-rutinoside), anthocyanins (cyanidin-glucoside), and triterpenoids (ursolic acid and oleanolic acid) (Table 7). In addition, Kiprovski et al. [62] found that cultivated elderberries had a higher content of phenolics compared to wild edible plants.

## 3. Health Benefits and Applications of Walnut By-Products (*Juglans regia* L.) and Elderberry (*Sambucus nigra* L.) Extracts

### 3.1. Health Benefits and Applications of Walnut By-Products (Juglans regia L.) Extracts

The walnut is a crop of great economic interest. Walnut trees are among the most important hardwood species in the northern hemisphere from an ecological and economic point of view. They are mainly cultivated for timber and nut production but are also attractive ornamental trees in parks. All the component parts are used in various industrial branches, including the food industry. The tasty part of the fruit (the kernel) is eaten fresh or roasted and alone or mixed with other edible products. Abdulwahid et al. [47] have shown that the walnut shell is a low-cost and abundant raw material, making it an attractive option for producing building materials. This can help to reduce the cost of construction and to make environmentally friendly building materials more accessible. According to Fordos et al. [48], the walnut shell is extensively used in agriculture, industrial fields, medicine, cosmetics, and environmental fields. This by-product can be a sustainable, ecological, and low-cost alternative to expensive toxic chemicals.

The benefits of the walnut, due to the presence of numerous bioactive compounds such as polyphenols, flavonoids, naphthoquinones, tannins, tocopherols, fatty acids, alkaloids, etc., are reflected not only on our health (with antibacterial, antioxidant, and anti-inflammatory benefits) but also on plants (the phytosanitary properties—antifungal and insecticidal). Like other medicinal plants, the walnut can be used due to its phytosanitary properties in the form of biopesticides, constituting a safe and cheap alternative compared to synthetic products that can be unfriendly to the environment.

#### 3.1.1. Antibacterial Activity

Studies have shown that all parts of the *J. regia* plant have antibacterial properties with beneficial effects on the human body. The green shell extracts of *J. regia* possess a good antibacterial activity on the species *E. coli*, *B. subtilis*, *P. aeruginosa*, and *S. aureus* [68]. Jaiswal et al. [69] showed that juglone inhibits three key enzymes in *H. pylori*. Antibacterial properties were enhanced if the dried walnut shell was modified with silver nanoparticles [70]. The antibacterial activity also depends on the method of extraction, the type of solvent, and the mixing ratio [24,43]. An ethanolic extract with a concentration of 5 mg/mL showed a high antibacterial potential for *S. aureus*. A concentration of 10 mg/mL was effective against a Gram-positive species (*L. monocytogenes*) and a Gram-negative species (*E. coli*). At a concentration of 5 mg/mL, the inhibition rates of *S. aureus*, *B. subtilis*, and *E. coli* bacteria were 85.31–90.26%. Aqueous extracts inhibited Gram-positive bacteria (*P. aeruginosa*) at a concentration of 100 mg/mL and *S. aureus* at a concentration of 0.1 mg/mL [71]. The walnut shell and leaf extracts also had an antibacterial effect against *S. mutans* dental caries [72]. Gomes et al. [73] studied the antibacterial activity of *J. regia*, individually and combined with *Eucalyptus globulus* hydromethanolic extract, and highlighted that the combination of both extracts was most effective against *S. aureus* from cow’s milk. An increase in the antimicrobial effect on *E. coli*, *P. aeruginosa* and *A. baumannii* was observed when using a walnut leaf extract mixed with ZnO nanoparticles [74]. Other studies presented the influences of the concentration of the same type of extract and the nature of the solvent used for extraction on the antibacterial activity on Gram-positive bacteria such as *E. faecalis* and *L. monocytogenes* [37]; *S. epidermidis*; *B. subtilis*; *S. aureus* [75]; *B. cereus* [38]; *S. epidermidis* [39]; and *S. arizonae* [76]. Dolatabadi et al. [77] determined that the aqueous extract had a better inhibition on *P. aeruginosa*. Huo et al. [78] showed that the walnut root extract had a strong antibacterial effect against *P. aeruginosa*, *S. aureus*, *E. coli*, and *P. mirabilis*. Extracts from the kernel and peel were proven to have an inhibitory effect on the strains of *E. faecium*, *E. coli*, *K. pneumoniae*, *P. aeruginosa* [79], and coagulase-negative staphylococci (MIC 3.60–461.75 µg/mL) [80]. Kavuncuoglu et al. [81] explained that the mathematical model based on Artificial Neural Networks (ANN) can be used to determine the inhibition zone diameter of the walnut extract for twelve bacterial species. Duda-Seiman et al. [82] showed that the alcoholic extract of the walnut kernel had a strong antimicrobial effect on the bacteria *S. pyogenes*, *S. aureus*, and *P. mirabilis* and was completely ineffective at the concentration tested against the Gram-negative bacterium *P. aeruginosa*. On the other hand, the study of Perreira et al. explained that aqueous extracts at low concentrations (0.1–1 mg/mL) inhibit Gram-positive bacteria (*B. cereus*, *B. subtilis*, *S. aureus*), and at higher concentrations (10–100 mg/mL) they inhibit the Gram-negative ones (*P. aeruginosa*, *E. coli*, and *K. pneumoniae*) [83]. Alcoholic septum extracts, according to the study of Genovese et al. [50], exhibited a stronger antibacterial effect on Gram-positive bacteria (*S. aureus*, *S. epidermidis*, *E. faecalis*, and *E. faecium*) than on Gram-negative strains (*E. coli*, *K. pneumoniae*, *P. aeruginosa*, and *P. mirabilis*), a fact indicated by lower MIC values. Ethyl acetate extracts from the walnut bark presented a high degree of antimicrobial activity for *S. mutans* and *S. salivarius* [84]; the alcoholic extract seemed to be more effective for *S. aureus* bacteria and less for *S. typhi* (bacterium Gram-negative) [85]; the aqueous extract, compared to the acetonic one, had a stronger inhibitory effect on the growth of the oral microbial flora [86,87]; and the chloroformic extract showed in vitro antimicrobial activity against dental caries microorganisms (*S. mutans, S. sobrinus, A. viscosus*) [41], as well as for *S. aureus, E. coli*, and *P. aeruginosa* [88]. The alcoholic, acetonic, and benzene extracts with the minimum inhibitory concentration of 50 μg/mL to 300 μg/mL inhibited Gram-positive bacteria (*B. subtilis* and *S. aureus*) [89]. It was also shown that chloroformic and aqueous walnut extracts had microbicidal activity against air microorganisms and the leaf extract was very effective in treating skin acne against the *Propioni bacteria* [16].

#### 3.1.2. Antifungal Activity

Pathogenic fungi are responsible for crop damage. Attempts are being made to replace fungicidal treatments that use synthetic, polluting chemical substances with more environmentally friendly plant extracts. Studies were carried out on extracts of the woody and green shells of walnuts, and their antifungal activity on cultivated plants, vegetables, and fruits was observed [90,91,92].

In the case of walnut bark extracts, Upadhyay et al. [26] demonstrated that the methanolic extract had significant activity against *A. niger*, the acetonic extract significantly inhibited the growth of *A. alternata*, and the chloroformic extract inhibited *T. virens* and *F. solani*. Ameziane et al. [93] observed a complete inhibition of the mycelial growth of *G. candidum* on citrus with methanolic and chloroformic bark extracts at a 10% concentration (*w*/*v*). 

Bennacer et al. [40] presented that in vitro the tannic extract from walnut leaves showed a high antifungal activity against *A. terreus, A. ochraceus*, and *A. brasilliensis* with an inhibition percentage of 77% for a concentration of 40 mg/mL. Wianowska et al. [94] demonstrated that walnut green shell extracts contain a substance called juglone, which exhibits an inhibitory activity for *A. alternata* of 45%. Walnut extracts had little effect on the *R. solani* that affects the rice sheath [95].

#### 3.1.3. Insecticidal Activity 

More and more, plant extracts are used as an alternative to combat crop plant pests. Walnut extracts have insecticidal properties. Walnut leaves can be introduced as an effective insecticide against the rice weevil (*S. oryzae*). On different days, after treatment, a concentration of 0.1 g/mL of extract was the most effective (66.66%) against the rice weevil [96]. Aqueous extracts of walnut leaves at a concentration of 5% caused 100% mortality in the nematode species *H. bacteriophora*, *S. carpocapsae*, *S. feltiae*, *S. kraussei*, and *P. hermaphrodita* [97]. Walnut extracts prevented 79.94% of tomato moth (*T. absoluta*) oviposition at a concentration of 20% [98] and have shown greater insecticidal activity on *T. griseus* beetle larvae compared to the synthetic insecticide *Tanalith C* [99]. Nevertheless, it was ineffective against the bean weevil (*A. obtectus*) [100]. Islam and Widhalm [101] observed that juglone had toxic effects on phytophagous insects such as melon, pepper, and tomato aphids (*A. gossypii*); the cabbage moth (*Trichoplusia ni*); the corn moth (*H. armigera*); the red mite (*T. urticae*), the cabbage butterfly (*P. rapae*), and the vinegar midge (*D. melanogaster*). The WCPI (Walnut Cysteine Protease Inhibitor) that is isolated from walnuts can inhibit fungal proteases, demonstrating its biopesticide potential, being an alternative to many chemical pesticides [102].

### 3.2. Health Benefits and Applications of Elderberry (Sambucus nigra L.) Extracts

In Europe, elderberry fruits and flowers are used both in food and in folk medicine. The elderflowers can be used in the form of elderberry juice or elderberry syrup, and also in the form of tea, after drying. Although the flowers and berries of the elder have many health benefits, elder leaves, branches, and roots contain substances that are toxic to the body, so they should be avoided. Terzic et al. showed the potential of wine obtained from elderberries as a new food product [103]. Elderberries are used in the treatment of many diseases due to their antioxidant, antitumoral, immunostimulatory, antiallergic, and antiviral benefits; their impact on obesity and metabolic disfunctions; their antidepressant potential; and their antidiabetic and antibacterial properties [12,13,14,65]. 

Due to the bioactive compounds, in addition to the health implications, there is a growing interest in the use of the elderberry in various fields, such as as natural additives in the food industry or as biopesticides in the growth of crop plants. 

Antibacterial activity with action on the human body as well as phytosanitary activity with benefits on cultivated plants are presented.

#### 3.2.1. Antibacterial Activity

Studies have indicated that the elderberry exhibits an antibacterial action that is found in the flowers, fruits, and leaves. Ferreira-Santos et al. [65] showed that aqueous extracts of the elderflowers have an antimicrobial activity against the Gram-positive bacteria *S. aureus* and *S. epidermidis*, and Ramanauskiene et al. [104] proved that ethanolic extracts had in vitro antimicrobial effects against *S. aureus* and *B. cereus*. Álvarez et al. [105] investigated the antibacterial role of peptide extracts from the elderflowers against various Gram-negative bacteria (*A. salmonicida*, *F. psychrophilum*, *V. anguillarum*, and *V. ordalii*). Antolak et al. [106] tested the effect of the elderflower extracts on six strains of *A. lannensis* and *A. bogorensis* bacteria and observed that the culture medium in the presence of elderberry is less favorable to the bacteria. Pinho et al. indicated a reduced antibacterial action on *K. pneumoniae* [60]. Methanolic extracts of the elderflowers did not inhibit the growth of *B. subtilis, P. aeruginosa*, and *S. aureus*, whereas ethanol extracts slightly inhibited the growth of *S. aureus* but did not inhibit *E. coli* [107]. The addition of 10% and 20% of elderberry extract to a culture medium inhibited Gram-positive (*Streptococcus* group G and group C) and Gram-negative (*B. catarrhalis*) bacterial growth by 70 and 99%. An infusion of elderberry leaves presented an inhibitory effect on the growth of bacteria (*B. subtilis*, *B. megaterium*, *E. coli*, and *S. aureus*) and yeasts (*D. hansenii*, *C. shehatae*, and *C. tropicalis*). The antimicrobial activity of extracts from the flowers was higher compared to extracts from the fruits [18]. Elderflower extracts were more toxic to Gram-positive (*Staphylococcus* sp. and *B. cereus*) and Gram-negative (*S. poona* and *P. aeruginosa*) bacteria compared to fruit extracts [108]. Salamon et al. showed that elderberry anthocyanins had no antimicrobial activity on *S. aureus* and *E. faecalis* [109] and that extracts from the leaves and fruits (aqueous 16%, ethanolic 5%, and methanolic 10%) had no antibacterial activity on *Streptococcus* sp., *E. coli*, *P. aeruginosa*, *S. typhimurium*, *S. marcescens*, *P. vulgaris*, *E. cloacae*, and *K. pneumoniae* [110]. Elderberry extracts had less inhibition for Gram-negative (*S. typhimurium*, *S. enteritidis*, and *E. coli*) than Gram-positive (*S. aureus*) [111]. Other studies indicated the antibacterial effect of elderberry extracts on Gram-positive (*E. faecalis*) and Gram-negative (*E. coli* and *P. fluorescens*) bacteria [112] and against *M. luteus*, *P. mirabilis*, and *P. fragii* bacteria [58]. Elderberry extracts (0.625–15 μg/mL) also inhibited the growth of *B. subtilis, S. aureus*, *P. aeruginosa*, *S. typhi*, and *E. coli* bacteria [113]. Młynarczyk et al. observed that elderflower extracts had a greater antimicrobial effect against *P. aeruginosa*, aqueous extracts of elder leaves had moderate activity against *B. cereus* and *S. marces*, and the fruits presented a superior potential against the bacteria that cause respiratory tract infections (a concentration of 20% of the fruit extract inhibited the growth in bacteria by 99%) [59]. Kačaniová et al. [114] and Allison et al. [115] established that the extracts from elder leaves (8–10 μg/mL) had an effect against the bacteria *S. enterica* and *S. pneumoniae*, but they did not show inhibitory activity against the growth of *E. coli*.

#### 3.2.2. Antifungal Activity

Rodino et al. demonstrated that the ethyl alcohol extract obtained from dried elderberry fruits showed the most intense inhibitory activity against the in vitro growth of *P. infestans* (potato mealybug) compared to the extracts from the elderflowers and fresh fruits, respectively [116]. Puia et al. established that an 8% ethanol extract of elderberry was effective against *Chaetomium* with a percentage of 96% and *Penicillium* sp. with a percentage of 85.69%, but was ineffective against *Fusarium* sp. [117].

#### 3.2.3. Insecticidal Activity 

In the literature, insecticidal activity has been mainly studied on extracts from elder leaves. Jankowska and Wojciechowicz-Żytko showed that aqueous extracts from the elderberry had insecticidal effects on cabbage aphids, with a percentage of 83.34% [118]. Elderberry extract produced a 20% mortality on *T. castaneum* (the reddish flour beetle) at a concentration of 100 mg/2 mL [119]. The insecticidal effect against the horn fly (*H. irritans*) was observed in vitro. Jovanović et al. observed that ethanolic extracts of elder leaves against the bean weevil were ineffective [100], and Ertürk determined that the toxic effect against the third and fourth stage larvae of the moth was low (60% of the larvae did not develop) [120]. 

## 4. Common Applications—Perspectives

Considering the data presented in this review, it is noted that some bioactive compounds are predominantly found only in walnuts belonging to different classes, such as juglone; α-hydrojuglone and derivatives (hydrojuglone β-D-glucopyranoside, hydrojuglone derivate pentoside, and hydrojuglone rutinoside); 1,4-naphthoquinone from naphthoquinones; regiolona, juglanone, and 4,5-dihydroxy-α-tetralone from tetralones; tannic acid from tannins; rosmarinic, 3-p-cumaroylquinic acids, and tyrosol from phenols and derivates; apigenin from flavonoids; and γ-tocopherol from tocopherols.

The antimicrobial action of the different parts of the walnut is demonstrated by the increased concentrations of naphthoquinones (juglone—4940 mg/100 g and 1,4-naphthoquinone—3680 mg/100 g, respectively). Juglone is reported to have antimicrobial, antifungal, oxidizing, and especially anti-proliferative actions [121,122]. At concentrations of 100–200 µg/mL in the growth medium, juglone irreversibly destroys the cell membranes for *Listeria* sp., which explains its bactericidal effect [123]. 1,4-Naphthoquinone exhibits antibacterial activity at concentrations of 2–10 µg/mL in the culture medium. A direct application on potato tubers in a concentration of 2 mg/mL inhibits the development of *E. carotovora* [124], a bacterium that produces black rot in a series of vegetables, such as carrots, red beets, onions, peppers, etc. The mechanism of the action of 1,4-naphthoquinone consists in the production of redox reactions that cause the appearance of reactive oxygen species, with a toxic effect on cell membranes [124].

Tetralones such as regiolone, juglanone, and 4,5-dihydroxy-α-tetralone also show antibacterial and antifungal activity against pathogens such as *E. coli*, *S. aureus*, *A. niger*, and *C. albicans* [125].

From the class of flavonoids, the walnut has significant contents of apigenin, which also exhibits antimicrobial actions specific to flavonoids in general [126].

Tannic acid presents bacteriostatic and antifungal actions on pathogens such as *L. monocytogenes* [127], *E. coli*, *Salmonella*, *S. aureus*, *S. epidermidis*, *C. albicans*, and *P. aeruginosa* [128].

Rosmarinic, 3-p-cumaroylquinic acids, and tyrosol are included in the class of phenols and their derivatives. Numerous studies report that rosmarinic acid is a powerful antibacterial and antifungal agent, stopping the growth of pathogens such as *B. cereus*, *S. aureus*, and *E. coli* [129]. 

Similarly, bioactive compounds predominantly found only in the elderberry are quinic acid from phenols and derivatives; naringenin and rutin from flavonoids; cyanidin-glucoside from anthocyanins; oleanolic acid from triterpenoids; and sambunigrin from cyanogenic glycosides.

From the class of flavonoids, naringenin and rutin stand out with significant concentrations. These compounds demonstrate actions similar to compounds common to the walnut and elderberry, which could lead to a potentiation of the antimicrobial action.

In the composition of the elderberry, there are compounds from the class of triterpenoids, such as oleanolic acid. This compound has been reported to have an antibacterial and antioxidant action on pathogens like *B. cereus*, *S. aureus*, *E. coli*, *S. typhimurium*, and *C. albicans* [130].

The specialized literature reports the antifungal and antibacterial action of the elderberry, mentioning sambunigrin in the composition of this plant, but a direct correlation between the presence of this compound and these activities has not yet been shown [13,58].

Besides these major compounds, *Juglans regia* and *Sambucus nigra* have several common bioactive compounds (gallic acid, caffeic acid, p-coumaric acid, ferulic acid, vanillic acid, quercetin, (+) catechin, (-) epicatechin, ursolic acid, ellagic acid, chlorogenic acid, neochlorogenic acid, and β-sitosterol) in varying amounts that act on the same microorganisms. 

Gallic acid shows antifungal and antimicrobial activity [131]. It possesses antifungal action on *Alternaria* sp. [132], *Candida* sp. [133], *A. niger* [134], and *F. solani* [135] and bacterial activity on Gram-positive and Gram-negative bacteria [136]. Studies have indicated decreases of 33, 75, and 81% in the growth rate, depending on the concentration of gallic acid. Treatments with gallic acid can be applied to vegetables affected by diseases caused by these fungi. The action of gallic acid consists in improving the activity of chitinase and peroxidase, thus promoting plant growth [135]. 

Caffeic acid has antifungal, antibacterial, and antibiotic properties on some pathogens, both for plants and humans. It is used with an antifungal and antibacterial role for human pathogens such as *C. albicans*, *S. aureus*, and *E. coli* [137]. This study also presents that gallic and caffeic acids can be used to improve the activity of some antimicrobial drugs. Concerning the applicability in agriculture, caffeic acid has an antifungal action on *F. graminearum*, a pathogen found in gramineae and corn. Its high pathogenicity consists not only in the decrease of productivity but also in the accumulation of some β-type mycotoxins in the plant mass [138]. Practically, caffeic acid has a negative impact on fungal growth and mycotoxin production.

Like the previous compounds, p-coumaric acid is part of the class of phenols and is reported in the specialized literature to have an antifungal and antibacterial action, both on human and agricultural pathogens [139,140,141].

Ferulic acid, found in many plant sources in varying amounts, is another phenolic compound common to the walnut and elderberries. It was applied with good results in combating the proliferation of *Listeria* sp. and *Salmonella* sp., where its inhibitory effect consisted in blocking the bacterial peptidoglycan [142]. It also shows antifungal activity on species such as *Candida* sp. and *Fusarium* sp. [143].

The antifungal action of vanillic acid on *L. theobromae*, *C. gloeosporioides*, *F. oxysporum*, *P. parasiticum*, *P. italicum*, *A. niger*, *A. alternate*, and *Trichoderma* sp. recorded lower effects compared to ferulic acid and p-coumaric acid [144]. Regarding the antibacterial activity, vanillic acid influences both human bacterial pathogens and those from agriculture or food.

Another compound common to the walnut and elderberry that has attracted attention is quercetin, a compound from the class of flavonoids. Jaisinghani reports the antibacterial action of quercetin on *S. aureus*, *E. coli*, *S. flexneri*, *P. vulgaris*, *P. aeruginosa*, and *L. casei* var. *Shirota* via the broth dilution method. The quercetin inhibited the action of these bacteria in a range of 20–500 µg/mL [145]. Numerous authors have reported the inhibitory effect of quercetin on *Candida* sp., *Aspergillus* sp., and *Fusarium* sp. [146,147,148]. From the class of flavonoids, the two species have significant contents of catechin and epicatechin with antimicrobial activity specific to flavonoids in general [126].

Ursolic acid, which is part of the class of triterpenes, also has antifungal and antibacterial action [130,149].

Ellagic acid, which belongs to the class of tannins, exhibits antibacterial and antifungal activity. Extracts in ethanol and chloroform have an inhibitory effect on *B. subtilis* and *S. aureus* [150]. The inhibition of pathogenic bacteria consists in the anti-enzymatic and antioxidant action [151].

Chlorogenic acid has a similar action to caffeic, rosmarinic, and ellagic acids with an antifungal and antibacterial role for human pathogens such as *C. albicans*, *S. aureus*, and *E. coli* [137,152].

β-Sitosterol, a compound from the phytosteroids class, in addition to other actions, possesses antifungal and antibacterial activities [153].

Considering the structure–biological activity relationship of the compounds, stud-ying the two plants together can lead to finding a synergy between them by cumulating the bioactive effect of all the compounds, in order to find inexpensive and non-polluting alternatives for both human and plant health.

## 5. Conclusions

Taking into account the areas cultivated with walnuts and the adaptability of the elderberry (in the spontaneous flora) in the eastern part of Romania, the two crops could constitute a continuous raw material for the extraction of bioactive compounds. Most of the compounds of the two plants have antifungal and antibacterial effects on both plant and human pathogens. This aspect leads to the idea of using them as enhancers for some medicines as well as biopesticides in the context of promoting environmentally friendly technologies and products. 

From the presented chemical compositions for both the walnut and the elderberry, it appears that all their components are rich in bioactive compounds. Both the elderberry and especially the walnut have been intensively studied for their individual, multiple biological activities both for human health and phytosanitary properties.

This review particularly highlights the antimicrobial activity on some pathogenic microorganisms that manifest their action on plant, leguminous, and fruit crops, which raises the hypothesis of the separate or combined use of walnut and elderberry extracts to prevent or stop the proliferation of pathogens. From this perspective, we have already initiated a research series with the aim of verifying the combined action of the phytochemicals of the two species, in order to obtain additional benefits in anticipation of a synergistic effect.

## Figures and Tables

**Table 1:** Main bioactive compounds of walnut kernel.

Compounds Class	Compound Name	References
Tannins	Glansreginin A	[27]
	Ellagic acid derivatives	[27,29,30]
Flavonoids	Procyanidin dimer 1	[27,34]
	Procyanidin trimer	[27,29,30,34]
	Catechin	[27,34]
Hydroxybenzoic acids	Gallic acid	[27,29,30]
Hydroxycinnamic acids	3-*O*-p-Coumaroylquinic acid	[27,29,30,34]
	Ferulic acid glucoside	
	Chlorogenic acid (3-*O*-Caffeoylquinic acid)	
Tocopherols	α-Tocopherol	[28,31,34]
	γ-Tocopherol	
	δ-Tocopherol	
Phytosteroids	Campesterol	[28,32,34]
	β-Sitosterol	
	Stigmasterol	
	Avenasterol	
Triterpenes derivatives	Cycloartenol	[33,34]
	2,4-Methyl cycloartenol	
Aliphatic alcohols	Docosanol	
	Tetracosanol	
	Hexacosanol	
Fatty acids	Linoleic acid	[34]
	Oleic acid	
	Linolenic acid	
	Palmitic acid	

**Table 2:** Main bioactive compounds of walnut leaf.

Compounds Class	Compound Name	References
Hydroxycinnamic acids	Neochlorogenic acid (5-*O*-Caffeoylquinic acid)	[35,36,37,38,39,40,42]
	3-p-Cumaroylquinic acid	
	Ferulic acid	
	Caffeic acid	
	p-Coumaric acid derivatives	
Flavonoids	(+) Catechin	
	(-) Epicatechin	
	Santin	
	Myricetin derivatives	
	Quercetin derivatives	
	Quercetin	
	Kaempferol derivatives	
Naphthoquinones	Juglone (5-Hydroxy-1,4-naphthoquinone)	[34,35,41,42]
	Hydrojuglone	
	Hydrojuglone derivatives	
	1,4-Naphthoquinone	

**Table 3:** Main bioactive compounds of walnut shell.

Compounds Class	Compound Name	References
Tannins	Ellagic acid	[20,43,44,45,46,49]
	Tannic acid	
Naphthoquinones	Juglone	[20,43,44,45,46,48]
	3-Methoxy-juglone	
	3-Ethoxy-juglone	
	8-Hydroxyquinoline	
	1,4-Naphthoquinone	
	5,8-Dihydroxy-1,4-naphthoquinone	
	2-Hidroxi-1,4-naphthoquinone	
Naphthoquinone glycosides	1,4,5-Trihydroxynaphthalene-1,4-di-β-d-glucopyranoside	
	1,4,5-Trihydroxynaphthalene-1,5-di-*O*-β-d-glucopyranoside	
	1,4,8-Trihydroxynaphthalene-1-*O*-β-d-glucopyranoside	
Naphthalenone	(4*R*)-3,4-Dihydro-4-butoxy-5-hydroxy-naphthalen-1(2*H*)-one	
Tetralones	Regiolona	[20,43,44,45,46]
	5,8-Dihydroxy-4-methoxi-α-tetralone	
	4,5-Dihydroxy-α-tetralone	
	Sclerone	
	Juglanone	
Hydroxybenzoic acids	Gallic acid	[20,43,44,45,46,47,48,49]
	Vanillic acid	
	Syringic acid	
	2,3-Dihydroxybenzoic acid	
	Tyrosol	
Hydroxycinnamic acids	Ferulic acid	
	Sinapic acid	
	Rosmarinic acid	
Flavonoids	(+) Catechin	[20,43,44,45,46,48]
	Quercetin	
	(−) Epicatechin	[20,43,44,45,46,48]
	Myricetin	
	Apigenin	
	Rutin	
Phytosteroids	β-Sitosterol	[19,20,43,44,45,46,47,48]
	Stigmasterol	
	Daucosterol	
	Campesterol	
Triterpenoids	Olenolic acid	[20,43,44,45,46,48]
	Oleanolic acid	
	Corosolic acid	
	Ursolic acid	
Sesquiterpenes	(+) Dehydrovomifoliol	
	Blumenol A	
Vitamins	Ascorbic acid	[20,49]
	α-Tocopherol	

**Table 4:** Main bioactive compounds of walnut septum.

Compounds Class	Compound Name	References
Hydroxybenzoic acids	Gallic acid	[50,51,52,53,54]
	Protocatechuic acid	
	Vanillic acid	
Tannins	2-Galloyl hexose	[50,51,52,53]
	Ellagic acid	
	Ellagic acid hexoside	
Flavonoids	Epigallocatechin	[50,51,54]
	Catechin	
	Epicatechin	
	Epigallocatechin gallate	
	Quercetin-3-*O*-glucoside	
	Quercitrin (Quercetin-3-*O*-rhamnoside)	
Hydroxycinnamic acids	p-Coumaric acid	[50,51,52,53,54]

**Table 5:** Main bioactive compounds of elderberry leaves.

Compounds Class	Compound Name	References
Hydroxycinnamic acids	5-p-Coumaroylquinic acid	[12,13,14,18,58,59,60,61,62]
	Caffeic acid	
	Chlorogenic acid	
	Neochlorogenic acid	
Flavonoids	Rutinoside-3-izorhamnetin	
	Rutinoside-3-kaempferol	
	Quercetin acetyl glucoside	
	Quercetin-rutinoside	
Anthocyanins	Cyanidin-3-p-coumaroyl sambubioside	
	Cyanidin-glucoside	
	Pelargonidin-rutinoside	
Cyanogenic glycosides	Sambunigrin	[58,59]

**Table 6:** Main bioactive compounds of elderberry flowers.

Compounds Class	Compound Name	References
Phenols and derivatives	p-Hydroxybenzoic acid	[18,58,64,65]
	Protocatechuic acid	
	Gentisic acid	
	p-Coumaric acid	
	Quinic acid	
	Ferulic acid	
Flavonoids	Naringenin	[58,60,64,65]
	Catechin	
	Epicatechin	
	Quercetin	
	Isorhamnetin	
	Neochlorogenic acid	
	Kaempferol-3-*O*-glucoside	
	Quercetin-3-*O*-hexoside	
	Kaempferol	
	Rutin	
Tannins	Ellagic acid	[18,60,64]
Terpenes	Ursolic acid	[64]

**Table 7:** Main bioactive compounds of elderberries.

Compounds Class	Compound Name	References
Phenols	Chlorogenic acid	[12,13,14,18,60,62,66,67]
	Neochlorogenic acid	
	4-Caffeoylquinic acid	
Flavonoids	Izorhamnetin-3-glucoside	
	Izorhamnetin-3-rutinoside	
	Quercetin-acetyl-hexoside	
	Quercetin-glucoside	
	Quercetin-rutinoside	
Anthocyanins	Cyanidin-sambubioside	
	Cyanidin-diglucoside	
	Cyanidin-glucoside	
	Cyanidin-3-p-coumaroil sambubioside-5-glucoside	
Fatty acids	Hexadecenoic acid	
	Octadecanoic acid	
	Eicosanoic acid	
Phytosterols	Campesterol	
	Stigmasterol	
	β-Sitosterol	
Triterpenoids	β-Amyrin	
	Oleanolic acid	
	Ursolic acid	

## Data Availability

Not applicable.
